# A male infant had subdural effusion and paroxysmal supraventricular tachycardia during the febrile episode of Kawasaki disease: a case report and literature review

**DOI:** 10.1186/s12887-016-0606-x

**Published:** 2016-05-28

**Authors:** Chia-Pei Chou, I-Chun Lin, Kuang-Che Kuo

**Affiliations:** 1 Department of Family Medicine, Kaohsiung Chang Gung Memorial Hospital and Chang Gung University College of Medicine, No.123, Dapi Rd., Niaosong Dist., Kaohsiung City, 833 Taiwan ROC; 2 Department of Pediatrics, Kaohsiung Chang Gung Memorial Hospital and Chang Gung University College of Medicine, No.123, Dapi Rd., Niaosong Dist., Kaohsiung City, 833 Taiwan ROC

**Keywords:** Infant, Kawasaki disease, Subdural effusion, Paroxysmal supraventricular tachycardia, Aspirin

## Abstract

**Background:**

Kawasaki disease is an acute, febrile, self-limiting, inflammatory systemic vasculitis seen in early childhood, most commonly in those below 5 years of age. In Kawasaki disease, the coronary arteries are most commonly affected, which may lead to asymptomatic coronary artery ectasia or formation of an aneurysm. Paroxysmal supraventricular tachycardia(PSVT) is a severe and rare cardiovascular complication of Kawasaki disease. A case of Kawasaki disease presenting with unusual findings, including subdural effusion and PSVT is reported.

**Case presentation:**

This is a 4-month-10-day-old boy presents with anterior fontanelle bulging and moderate bilateral subdural effusion at the acute stage of Kawasaki disease and PSVT at the subacute stage of Kawasaki disease. The subdural effusion was resolution after intravenous immunoglobulin(IVIG) administration. And the PSVT was subsided after administered 3 doses of adenosine, 1 dose of amiodarone loading and Propranolol twice per day use. At 1-year follow-up has made a complete recovery with no arrhythmia episodes, developmental effects or abnormal neurologic findings.

**Conclusion:**

Subdural effusion in the acute stage of Kawasaki disease may be an inflammatory response. It may resolves spontaneously after anti-inflammatory treatment such as IVIG infusion. PSVT is a severe cardiovascular complication of Kawasaki disease. In those who taking aspirin, we need to carefully observe the heart rhythm and PSVT side effects, especially in the first month.

## Background

Kawasaki disease is an acute, febrile, self-limiting systemic vasculitis seen in early childhood, most commonly in those below 5 years of age. This condition is the leading cause of acquired heart disease in children [1]. There are no specific biomarkers for the diagnosis of Kawasaki disease.

The diagnostic criteria of typical Kawasaki disease include fever lasting for 5 days in addition to 4 of the following 5 clinical features: oropharyngeal changes; non-purulent conjunctivitis; acute cervical lymphadenopathy with lymph node diameter > 1.5 cm; peripheral extremity changes; or a generalized polymorphous rash [2, 3].

In Kawasaki disease, the coronary arteries are most commonly affected, which may lead to asymptomatic coronary artery ectasia or formation of an aneurysm. Rarely, Kawasaki disease may cause myocarditis, pericarditis, or lead to arrhythmias. Ventricular arrhythmia is also an unusual finding in Kawasaki disease in the past case presentation or literature review. Kawasaki disease is a systemic inflammatory disease commonly associated with thrombocytosis, especially after the first week of illness [4]. The incidence of Kawasaki disease with neurological complications is approximately 1.1-3.7 %. Subdural fluid collection is rarely described in association with Kawasaki disease in the previous literature.

Full doses of intravenous immunoglobulin (IVIG) are the mainstay of treatment. Early administration of IVIG may reduce the risk of cardiac involvement to 5 % [5]. During the clinical course, close monitoring of cardiovascular function and coronary artery diameter is necessary. For coronary artery abnormalities, aspirin is part of the standard treatment [5]. In most children, the heart problems subside after 5 or 6 weeks without lasting damage. However, in some children, the coronary arteries are damaged permanently.

## Case presentation

The 4-month-10-day-old boy presented with intermittent fever for 2 days and he was taken to emergency department on February 16, 2014. He had received a 10-valent pneumococcal vaccine (PCV 10), 2 days prior to his visit, in a local clinic and did not have a fever or throat injection at the time. After admission, physical examination revealed injected throat and tonsils, coarse breathing sounds, polymorphous rashes over the trunk, anterior fontanelle bulging, and poor activity while febrile. Laboratory examination revealed leukocytosis (WBC: 28300/uL), segmented predominance (segment: 67 %) and an elevated CRP level (CRP: 69.4 mg/L). Urine analysis showed mild pyuria (WBC: 12/uL) and ketones (ketone: 40 mg/dL). He was admitted to the hospital for suspected bacterial meningitis, and antibiotic therapy with cefotaxime was started. However, his parents declined our suggestion for cerebrospinal fluid (CSF) tapping.

**Fig. 1:**
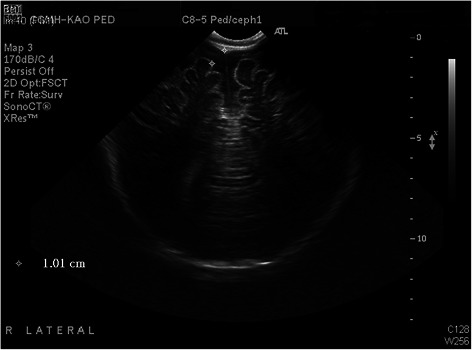
Brain ultrasound. Initial brain ultrasound on admission with moderate subdural fluid collection(1.01 cm)

Persistent intermittent fever was noted on February 17, 2014. In addition, he presented with injected and fissured lips, a strawberry tongue, erythema and edema over palms and soles, and bilateral bulbar conjunctival injection. The echocardiography was performed for possible Kawasaki disease on February 17, 2014 and revealed the z-score of the left coronary artery and right coronary artery was 1.25 and 1.83, no coronary artery dilatation complication. Swelling with erythematous change over the Bacillus Calmette-Guérin vaccine (BCG) injection site was noted on February 18, 2014. Brain ultrasound (Fig. 1) was also performed for anterior fontanelle bulging on February 18, 2014 and revealed moderate subdural fluid collection.

Due to persistent intermittent fever for 5 days, on February 18, 2014, IVIG therapy (IVIG 2 g/kg administered over 12 h) was prescribed from that afternoon until the next morning. Aspirin 30 mg once per day was prescribed starting February 19, 2014. Fever subsided gradually from February 21, 2014, following which low-dose aspirin was continued. The symptoms of polymorphous rash, injected and fissured lips, and BCG injection site swelling showed improvement. The patient was discharged without any discomfort or fever on February 25, 2014.

**Fig. 2:**
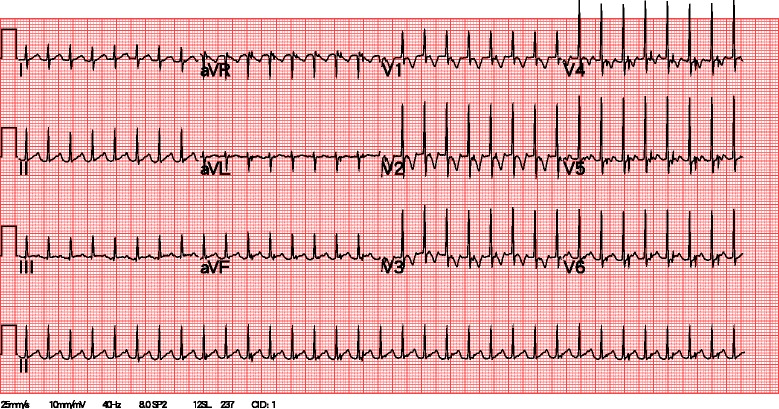
Electrocardiogram. Initial ECG with paroxysmal supraventricular tachycardia

**Fig. 3:**
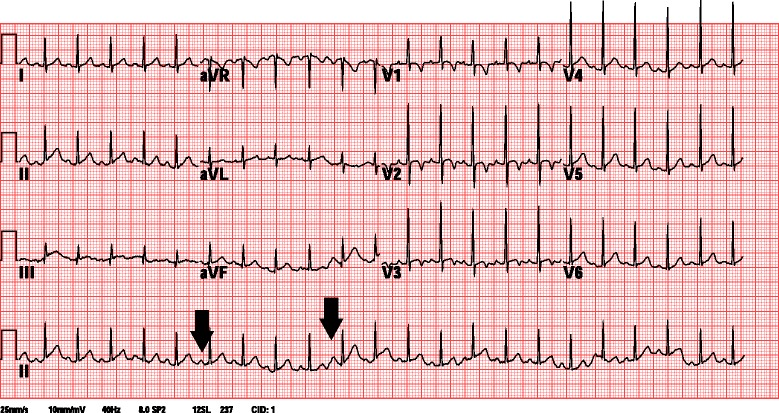
Electrocardiogram. ECG with normal sinus rhythm after 3 doses of adenosine, amiodarone loading dose of 5 mg/kg, and Propranolol. Arrow mark: P waves

The patient experienced a fever of 38.9 °C again on March 5, 2014 in the early morning hours, and then subsided spontaneously. In addition, a mildly injected lip and distal limbs swelling without desquamation were noted. Tachyarrhythmia (heart rate > 200 beats/min) was present at the afternoon. Electrocardiogram (ECG) (Fig. 2) showed PSVT. The echocardiography revealed tachycardia with the z-score of the left coronary artery and right coronary artery was 2.5 and 1.94, no evidence of coronary artery dilatation, no ischemic change, and no other abnormal findings. Laboratory examination showed leukocytosis (WBC: 17300/uL), thrombocytosis (platelet: 820000/uL), an elevated CRP level (CRP: 117.4 mg/L), an elevated ESR level (ESR: 108 mm/h), and normal cardiac enzymes. The ECG monitor persistently showed PSVT, even when the patient did not have a fever and was not crying. We administered 3 doses of adenosine (1 mg, 1 mg, and 2 mg intravenous push). His heart rate was lowered to 60 beats/min with adenosine administration, but was refractory soon after crying and being in an irritable mood. Therefore, we initiated an amiodarone loading dose of 5 mg/kg (40 mg in 20 ml of D5W) for 1 h. Following this patient’s condition improved and was relatively stable; the ECG monitor showed tachyarrhythmia (heart rate 150–160 beats/min) with the P wave. Propranolol 2.5 mg twice per day was also prescribed for control of the arrhythmic heart rate, after which, tachyarrhythmia was improved with a baseline heart rate of 125–135 beats/min. The repeat ECG (Fig. 3) showed a normal sinus rhythm with normal PR and QT intervals and no delta waves.

**Fig. 4:**
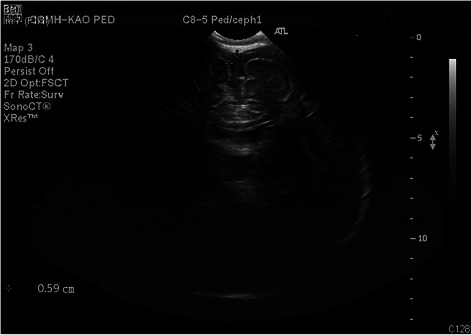
Brain ultrasound. Follow-up brain ultrasound 2 weeks after IVIG administration showing mild subdural fluid collection(0.59 cm)

Brain ultrasound (Fig. 4) performed on March 6, 2014 revealed mild subdural fluid collection. Blood, urine, and CSF cultures were sterile. Because CSF microscopic analysis showed no pleocytosis, with normal sugar, protein, and chloride levels, bacterial meningitis was less likely. Desquamation on tips of fingers and toes appeared on March 8, 2014. The echocardiography performed on March 10, 2014 revealed a normal echocardiogram with normal coronary arteries. We stopped propranolol use on March 14, 2014. The patient was discharged on March 15, 2014, as his general condition had improved, and his vital signs were relatively stable.

The patient had regular follow-up examinations at the pediatric outpatient department. The echocardiography revealed a normal echocardiogram and normal coronary arteries. ECG also showed a normal sinus rhythm. He had taken aspirin for 2 months, which was discontinued on April 21, 2014. Brain ultrasound revealed complete resolution of the subdural effusion. No more arrhythmia episodes, developmental effects or abnormal neurologic findings were detected at the 1-year follow-up.

## Discussion

### Subdural effusion in Kawasaki disease

The incidence of Kawasaki disease with neurological complications is approximately 1.1-3.7 %. Nervous system complications previously reported include seizure, facial nerve palsy, meningoencephalitis, hemiplegia, ataxia, chorea, ischemia, abnormal vision, disturbed consciousness, behavioral changes, sensorineural hearing loss, and monocyte-predominant pleocytosis in CSF [6, 7]. Subdural fluid collection is rarely described in association with Kawasaki disease. Only 7 equivocal cases of extracerebral fluid collection or subdural effusion in the acute stage of atypical Kawasaki disease have been reported in the literature [8, 9].

Aoki et al. [8] reported the case of a 6-month-old girl in the acute stage of Kawasaki disease, who presented with retinal hemorrhage complications and bilateral subdural fluid collection with intracranial hypertension. The subdural effusion with intracranial hypertension was controlled using a subdural tap and glycerol administration. Additionally, a computerized tomography (CT) scan confirmed reduction of subdural fluid collection.

Bailie et al. [9] described the case of a 6-month-old boy with possible Kawasaki disease, who had complications related to bilateral subdural collection and prolonged right-sided seizure. He also presented with tachycardia (approximately 120–150 beats/min). The patient had an amazing response to administration of IVIG (2 g/kg) and high-dose aspirin (100 mg/kg/day in 4 divided doses). CT scan showed complete resolution of the subdural collection at the 6-months follow-up.

Our patient is a typical case of Kawasaki disease with moderate bilateral subdural effusion and is the youngest one of all previously reported cases in previous literature. However, our patient only presented with anterior fontanelle bulging and poor activity when febrile, without any specific neurological signs or encephalopathy symptoms. This patient underwent only IVIG administration as an anti-inflammatory measure in the acute stage of Kawasaki disease. Brain ultrasound revealed a definite reduction in the volume of subdural effusion 2 weeks post-presentation and IVIG infusion. There was no any lasting damage, and this child demonstrated normal development at the 1-year symptom-free follow-up.

### PSVT in Kawasaki disease

The cardiovascular complications of Kawasaki syndrome commonly include symptoms related to coronary artery dilation, coronary aneurysm, myocarditis, or myocardial ischemia. Ventricular arrhythmia is an unusual finding and rare complication in the acute phase of Kawasaki disease; this sign is independent of coronary artery involvement in Kawasaki disease [10].

Haney et al. [11] reported a 2.5-year-old child with ventricular arrhythmia in the acute stage of Kawasaki disease. The arrhythmia was resolved after empirical treatment with oral steroids.

Seymour et al. [12] described the case of a 12-year-old girl with medical history of Kawasaki disease diagnosed at the age 2 years and who received appropriate treatment for this condition. The patient presented with supraventricular tachycardia (SVT) at 180 beats/min. Intravenous access and vagal maneuvers were performed to abate the SVT without success. After 2 doses of adenosine was administered by rapid intravenous bolus, the heartbeat decreased to a sinus rhythm of 88 beats/min. No further damage was noted during an outpatient follow-up.

Our patient is a typical and rare case of Kawasaki disease with PSVT. Tachyarrhythmia (heart rate > 200 beats/min) and PSVT were presented after admission in the subacute stage of Kawasaki disease, and our patient had taken aspirin for only 2 weeks. It was difficult to control his heart rate. We had attempted 3 doses of adenosine (1 mg, 1 mg, and 2 mg intravenous push), and amiodarone loading dose of 5 mg/kg (40 mg in 20 ml of D5W) for 1 h to control the PSVT. Propranolol 2.5 mg twice per day was added to control the arrhythmia. There were no signs of any defects or damage on the ECG and echocardiography at the 1-year follow-up.

### IVIG in the subacute stage of Kawasaki disease

The clinical presentation of Kawasaki disease is conventionally divided into 4 stages: acute, subacute, convalescent and chronic. The subacute stage of Kawasaki disease begins when the fever has abated, and it may last for 4–6 weeks. The rate of sudden death is the highest in the subacute stage. Persistence of fever beyond 2–3 weeks in the subacute stage of Kawasaki disease may be an indication of recrudescent Kawasaki disease or persistent Kawasaki disease. This condition requires secondary IVIG therapy [13].

On the other hand, we have defined recurrent Kawasaki disease as a separate episode fulfilling Kawasaki disease criteria after an earlier occurrence has fully resolved, typically at least 2 months later [13]. The recurrence rate was 6.89 per 1000 person-years, with a high incidence within 12 months following the first episode [14]. Recurrent Kawasaki disease requires reevaluation and retreatment with IVIG and aspirin. Patients with recurrent Kawasaki disease are at an increased risk for developing cardiac sequelae.

In our patient, the secondary fever episode subsided after treatment with antipyretics, and the fever episode duration was less than 24 h. It is obvious that the secondary fever episode in our patient was not a case of recrudescent or recurrent Kawasaki disease. Therefore, our patient did not need secondary IVIG infusion or retreatment. The only intervention needed was symptom-control and monitoring of further complications of Kawasaki disease.

## Conclusions

We hereby present a young infant with IVIG-responsive subdural effusion and difficult-to-control PSVT complications due to Kawasaki disease.

Subdural effusion in the acute stage of Kawasaki disease may be an inflammatory response. Administration of IVIG relieves acute inflammation. As in our case, there is no need for a subdural tap or any surgical intervention; the subdural effusion resolves spontaneously after anti-inflammatory treatment such as IVIG infusion. Additionally, there is no any lasting defect or damage in patients with Kawasaki disease. PSVT is a severe cardiovascular complication of Kawasaki disease and may be related to sudden death or lasting damage in the case of incomplete symptom control or inadequate treatment. In those who taking aspirin, we need to carefully observe the heart rhythm and PSVT side effects, especially in the first month. A second fever episode in a patient with Kawasaki disease should be differentially diagnosed. If the fever occurs during the subacute stage of Kawasaki disease and persists less than 2–3 weeks, it is not a case of recrudescent Kawasaki disease or recurrent illness. Treatment with secondary IVIG therapy or corticosteroids is therefore not requires.

## Abbreviations

BCG: Bacillus Calmette-Guérin vaccine
CSF: cerebrospinal fluid
CT: computerized tomography
ECG: electrocardiogram
IVIG: intravenous immunoglobulin
PSVT: paroxysmal supraventricular tachycardia
SVT: supraventricular tachycardia
